# A Global Environmental Scan of Competencies for Mobility Assistive Product Provision

**DOI:** 10.3390/healthcare12171747

**Published:** 2024-09-02

**Authors:** Louise Puli, Libby Callaway, Christina L. Ekegren, Natasha Layton

**Affiliations:** 1 Rehabilitation, Ageing and Independent Living (RAIL) Research Centre, Monash University, Melbourne, VIC 3199, Australia; libby.callaway@monash.edu (L.C.); christina.ekegren@monash.edu (C.L.E.); natasha.layton@monash.edu (N.L.); 2 Occupational Therapy Department, Monash University, Melbourne, VIC 3199, Australia

**Keywords:** competency standards, frameworks, assistive products, mobility, standards, environmental scan, rehabilitation, assistive technology

## Abstract

Competencies defined in practice standards and frameworks promote safe and effective healthcare by underpinning training curricula and service standards. The provision of mobility assistive products involves a range of professionals, each guided by various standards and frameworks that define various competencies. This environmental scan aimed to identify global resources containing professional competencies applicable to mobility assistive product provision and to compare them against a gold standard. Competencies for mobility assistive products were found in 14 resources of diverse types. While there were similarities between competency standards, such as service steps, there were also significant differences, such as minimum education levels and competency domains. This environmental scan highlights an opportunity for professionals providing mobility assistive products to collaborate and establish unified competencies. Standardizing these competencies could harmonize training, regulation, and service standards, thereby improving coordination and service quality. Additionally, this approach could serve as a model for developing standardized competencies for other assistive products, such as hearing and vision aids, enhancing outcomes across various assistive technology types.

## 1. Introduction

Globally, 2.5 billion people require assistive products to improve their independence, participation, and quality of life [1]. A significant proportion of the need relates to assistive products for mobility (hereby termed ‘mobility assistive products’) [1,2,3,4,5]. The WHO International Classification of Functioning, Disability, and Health (ICF) defines mobility as moving by changing body position or location or by transferring from one place to another, by carrying, moving, or manipulating objects, by walking, running, or climbing, and by using various forms of transportation [6].

Mobility assistive products optimize function and reduce disability in moving around [7,8,9,10]. Mobility is fundamental to a myriad of daily activities and community participation as evidenced by the prominence of mobility assistive products within the World Health Organization (WHO) Packages of Rehabilitation Interventions [9]. Details about mobility assistive product types, as classified by the International Standards Organization (ISO), are in Appendix A [11].

The WHO and UNICEF have highlighted that the current assistive technology workforce cannot meet the demand for mobility assistive products [1]. Given recent projections, which suggest that the number of people needing assistive products will increase to 3.5 billion by 2050, a significant gap in meeting future mobility assistive product needs is anticipated. To address this issue, the WHO and UNICEF Global Report on Assistive Technology calls for competency-based approaches to growing the assistive technology workforce, promoting its development, whilst maintaining service quality and safety [1]. This includes defining the common professional competencies (hereafter referred to as competencies)– that is, knowledge, skills, and attitudes—needed across professions. Once defined, competencies can be found in standards and framework documents that guide practice, support services, and underpin training, education, and regulation [12]. The call for competency standards is echoed in other reports, including the WHO Global Report on Health Equity for Persons with Disabilities, WHO’s Rehabilitation 2030 Initiative, and in the broader literature, which suggests that assistive technology competencies need to be systematically embedded into broad health curricula, such as that for rehabilitation, noncommunicable diseases, and caring for older people [13,14,15,16]. 

The lack of a defined set of competencies for mobility assistive product provision across various professions prevents a cohesive approach to service delivery and risks inconsistent service quality [14,17]. It also leaves uncertainty about the minimum level of education required to safely and effectively provide mobility assistive products and the necessary content of any related curricula or training program. In the absence of standardized competencies, each profession that provides advice on mobility assistive products likely follows its own guidelines. This lack of uniformity makes it uncertain whether all professions approach the provision of these products in a consistent manner, potentially leading to gaps in care and varying levels of service quality for assistive product users. 

Whilst there are no international competency standards for assistive product provision, the WHO published a Rehabilitation Competency Framework (WHO RCF) in 2021 [10]. The intention of this work was to provide an adaptable model as a foundation for creating additional frameworks for various specializations. The WHO RCF is therefore well placed to serve as a comparison for existing assistive product-related competency standards. 

Given the critical role that mobility plays in the independence and quality of life for users of assistive products and the significant risks associated with improper provision, this research presents an environmental scan of global competency standards and frameworks in professions related to mobility assistive products. For the purpose of this research, ‘global’ standards and frameworks were defined as internationally applicable resources that outline the essential skills, knowledge, and behaviors required for effective assistive product provision. The primary objectives of this scan were to identify and describe the existing global competency standards and frameworks applicable to mobility assistive product provision and to compare these against the WHO RCF.

## 2. Materials and Methods

Professional competencies are typically detailed in documents published by professional bodies within the grey literature, rather than in the academic literature. Consequently, an environmental scan was deemed the most appropriate methodology for this research [18,19]. The environmental scan consisted of an internet search to identify global standards documents in the area of mobility assistive products (Step 1) and a consultation with global professional bodies for further information (Step 2).

**Step 1: Internet Search for Global standards**

Prior to commencing the internet research, a protocol was developed a priori by the research team with input from a University medical librarian. The search strategy (Appendix B) was refined over several rounds of testing, with the finalized protocol then registered on Open Science Framework in December 2023 before the environmental scan commenced [20]. 

An advanced Google search was performed in February 2024 using specified keywords to identify relevant standards resources in the grey literature, including terms relating to assistive products, competency standards, and mobility. Geolocation settings were disabled in Google and Google Chrome to ensure the search was global and not influenced by the researcher’s location. Additionally, a second search was performed on the Overton platform, a searchable index for policy documents using the same search terms [21]. The Overton platform was chosen because it specializes in indexing policy and the grey literature, which are crucial for capturing non-academic authoritative resources relevant to competency standards. This allowed us to access a broader range of documents that are not typically found through standard academic searches, providing a more comprehensive view of the existing frameworks and guidelines.

### Inclusion Criteria

Since there is no internationally accepted list of professions that provide mobility assistive products, this environmental scan included the professions listed as assistive technology direct service personnel in both the WHO and UNICEF Global Report on Assistive Technology as well as the WHO RCF (Table 1), being audiology, occupational therapy, prosthetics and orthotics, physiotherapy, and speech and language therapy, medical, nursing and psychology rehabilitation specialists, rehabilitation assistants, technicians, and community-based rehabilitation workers [1,10]. However, as this environmental scan focused on mobility assistive products, retrieved sources specific to professions with a focus on hearing (audiology), speech (speech and language therapists), and cognition (psychologists) were subsequently excluded. 

The included resources were those written in English and there were no restrictions on the publication year or document format. The search was limited to the first 200 results to ensure the most recent and pertinent resources were located from both platforms (Google and Overton). In cases where a professional body had released multiple versions of resources, only the most recent version was included. If a profession was represented by two different professional bodies, documents from both were included. If no documents were found for a particular professional group, the search did not extend further online; instead, the professional body was contacted directly as part of step 2.

**Step 2: Consultation with professional bodies**

In Step 2, the complete list of relevant international professional bodies was consulted (Table 1). Contact was initiated by the first author via email, using the contact information available on the website of each professional body. The email contact sought access to information on their current competency standards related to mobility assistive product provision and invited contributions of any new or recently updated resources. If a professional body did not respond, follow-up actions included subsequent emails 2 and 4 weeks after initial contact. The information received from the professional body was systematically recorded and incorporated with data collected in Step 1.

**Data extraction and synthesis**

Data were extracted from each electronic source that met inclusion criteria and entered into a data extraction table developed by the research team. The data extraction table was piloted independently by two researchers (L.P and N.L), with three included resources undergoing extraction by each independently [22]. The researchers then met to examine consistency and ensure consensus on data extraction. In cases of identified conflicts, two researchers discussed or consulted a third researcher in the team if the conflict persisted until a consensus was reached. Any necessary changes were then made to the data extraction table. 

Data were then extracted from each of the included resources by the first author and validated by a second researcher via a review of the original file source and data extracted.

Data extracted included the resource name, year of publication, languages available, an indication of global applicability and public accessibility, and the number of countries that had adopted the resource. 

To meet the second objective of comparing competency standards and frameworks to the WHO RCF, each identified resource was then compared against the WHO RCF domains of (i) practice, covering competencies related to the direct provision of services to clients; (ii) management and leadership relating to competencies related to collaborative and reflective practice; (iii) professionalism covering competencies related to compliance to ethics, standards, and guidelines; (iv) learning and development covering competencies related to ongoing personal and service development via learning and the application of new knowledge ensuring ongoing best practice, and (v) research, covering competencies related to the integration of evidence into practice. For comparison with the WHO RCF, additional items extracted from each resource included the international educational level stipulated, the definition of competency, the purpose of the resource, the evidence or procedures used to identify the listed competencies, the key competency domains, service steps, the levels of proficiency or skill specified, curriculum guidance given, and any mention of mobility and assistive products within each resource. The goal was to identify the degree of alignment with the WHO RCF, noting any significant gaps, similarities, or differences, which could inform potential areas for further development or harmonization.

## 3. Results

**Figure 1:**
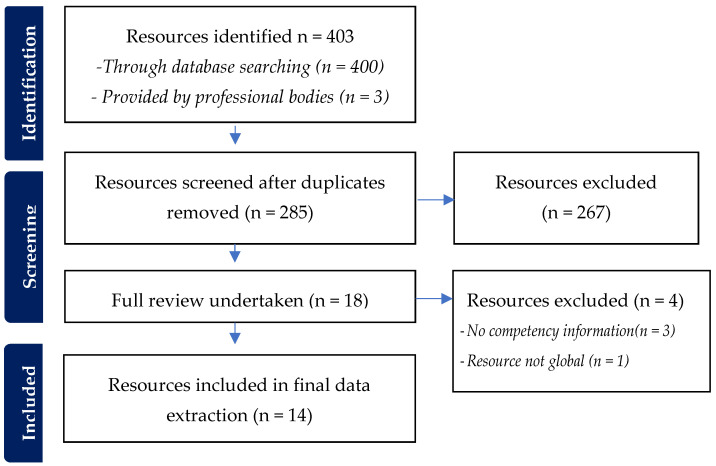
Search results.

The initial internet search yielded 400 resources, with three additional resources identified by contacting professional associations (Figure 1). These three additional resources, relating to wheelchair provision, were provided by the International Society of Wheelchair Professionals (ISWP). After removing duplicates, 285 resources were screened by title and description. Of these, 18 resources proceeded to full review. Further reasons for exclusion included resources not meeting the definition of being ‘global’ (as they specified applicability to a single country or region) and/or lacking any mobility assistive product competency information. Ultimately, 14 resources met the study inclusion criteria and underwent data extraction and synthesis.

### 3.1. Resource Types and General Features 

The 14 included resources were published between 2011 and 2023. Resources identified were able to be classified into five broad categories: (i) Service standard/guide (n = 5), (ii) Training resources (n = 3), (iii) Education standard (n = 3), (iv) Workforce frameworks (n = 2), and (v) Organizational manual (n = 1) and (6) Profession profile/description and scope of practice (n = 1) as displayed in Table 2.

Only half of the identified resources were published by known global professional bodies. These included the International Society of Prosthetics and Orthotics (ISPO), the International Society for Wheelchair Professionals (ISWP), World Physiotherapy (WP), and the World Federation of Occupational Therapy (WFOT). The remaining resources were provided by intergovernmental bodies (UNICEF and WHO), a government agency (United States Agency for International Development-USAID), a humanitarian organization (International Committee of the Red Cross-ICRC), and a training organization (Physiopedia). No resources were identified for organizations representing nursing, medicine, community health workers, or rehabilitation medicine. 

Both profession-specific and multidisciplinary resources were identified (Table 2). Profession-specific resources highlighted standards for individual healthcare professions. For instance, in 2021, WP published a framework for physiotherapist education. Similarly, in 2017, the WHO outlined the roles and scope of practice for biomedical engineers, and in 2017, ISPO established education standards for prosthetic and orthotic occupations. The WFOT in 2016 released minimum education standards for occupational therapists.

As examples of multidisciplinary resources, the WHO, ISWP, and ISPO together published wheelchair provision guidelines in 2023, targeting all professions that may provide wheelchairs. The WHO’s 2023 Global Competency and Outcomes Framework for Universal Health Coverage was found to address the health workforce broadly. 

Some resources were available in English and languages other than English [23]. As an example, the WFOT minimum standards for the education of occupational therapists were available in seven additional languages [23] and the WHO Mobility Assistive Products training module in 13 additional languages [24]. 

#### Focus of Resources

The identified resources demonstrated a diverse range of approaches and focuses. The only resource that focused exclusively on the topic of mobility assistive products was the WHO’s Training in Assistive Products (WHO TAP): Mobility Assistive Product Module. This introductory module is augmented by a range of specific modules addressing mobility, including walking aids [11].

Two other resources for wheelchair provision developed by WHO, USAID, ISPO, and ISWP focused on training wheelchair professionals and wheelchair provision standards. The WHO’s “Package of Interventions for Rehabilitation: Module 2: Musculoskeletal Conditions” was unique in taking a health condition focus and organizing the assistive products into separate musculoskeletal conditions. 

All other resources either addressed assistive products in general or included mobility-assistive products as a subsection of a broader range of assistive products and health services.

### 3.2. Mapping to the WHO Rehabilitation Competency Framework

#### 3.2.1. Minimum Education and Proficiency Levels 

ISPO’s Education Standards for Prosthetic/Orthotic Occupations was the only resource that linked to a global benchmark for the level of education required, citing alignment to the United Nations Educational, Scientific and Cultural Organization (UNESCO) Global National Qualification Framework [25]. ISPO’s resource also used the regional European Qualifications Framework (EQF) as an example. The ISPO specified that prosthetic and orthotic technicians should meet a minimum EQF level 4 (certificate level), associate prosthetists/orthotists should meet EQF level 5 (diploma level), and prosthetists/orthotists should meet EQF Level 6 (bachelor’s degree level) [26]. 

Three other profession-specific resources published by WHO, WFOT, and WP stated the minimum international education levels needed to enter their profession using common terminology, i.e., ‘bachelor’s degree’ [23], without linking to a benchmark. Supportive workforces were also described, including prosthetic/orthotic technicians and physiotherapy assistants, who were described as requiring ‘diploma’ qualifications, with no minimum qualifications set for the cadre of ‘physiotherapy helpers’ [27,28]. WP indicated that physiotherapists are required to have a bachelor’s degree or higher. WFOT in their minimum standards for the education of occupational therapists also upheld a bachelor’s degree as the minimum standard, and further to this, set an additional stipulation that occupational therapy training should occur ‘in higher education institutions) or equivalent’. For biomedical engineers, the Accreditation Board for Engineering and Technology accredits university programs in engineering at the levels of associate (EQF 5), bachelor’s (EQF 6), and master’s degree (EQF7).

#### 3.2.2. How the Competencies Were Established 

Like the WHO RCF, the introductory section of several resources described the methodology used to establish the competencies contained within them. Resources that included such details were the WHO global competency and outcomes framework for universal health coverage, WHO human resources for medical devices, the role of biomedical engineers, the WHO wheelchair provision guidelines, ISPO education standards for prosthetic/orthotic occupations, and the WHO package of interventions for rehabilitation [26,29,30,31]. The methods used to establish competencies included systematic literature reviews, interviews, and iterative Delphi processes. Other approaches, such as surveys, were also cited in some resources, for example, the WFOT minimum standards for the education of occupational therapists [23]. The remaining resources either provided no information or significantly less information regarding the method used to establish the described competencies.

#### 3.2.3. Structure: Domains, Activities, and Competencies

The education and workforce resources classified as frameworks (Table 2) were structured in a similar manner to the WHO RCF. They provided competency information organized under overarching ‘domains’ or areas of competency, followed by observable or measurable activities or performance indicators. 

In comparison to the five domains in the WHO RCF (practice; management and leadership; professionalism; learning and development; and research), the identified resources tended to have more domains (Table 3). The Global Competency Framework for Universal Health Coverage identified health worker competencies within six domains: people-centeredness, decision making, communication, collaboration, evidence-informed practice, and personal conduct. The WP education framework domains included physiotherapy assessment and intervention, ethical and professional practice, communication, evidence-based practice, reflective practice and life-long learning, quality improvement, and leadership and management. 

#### 3.2.4. Practice Activities/Steps

**Figure 2:**
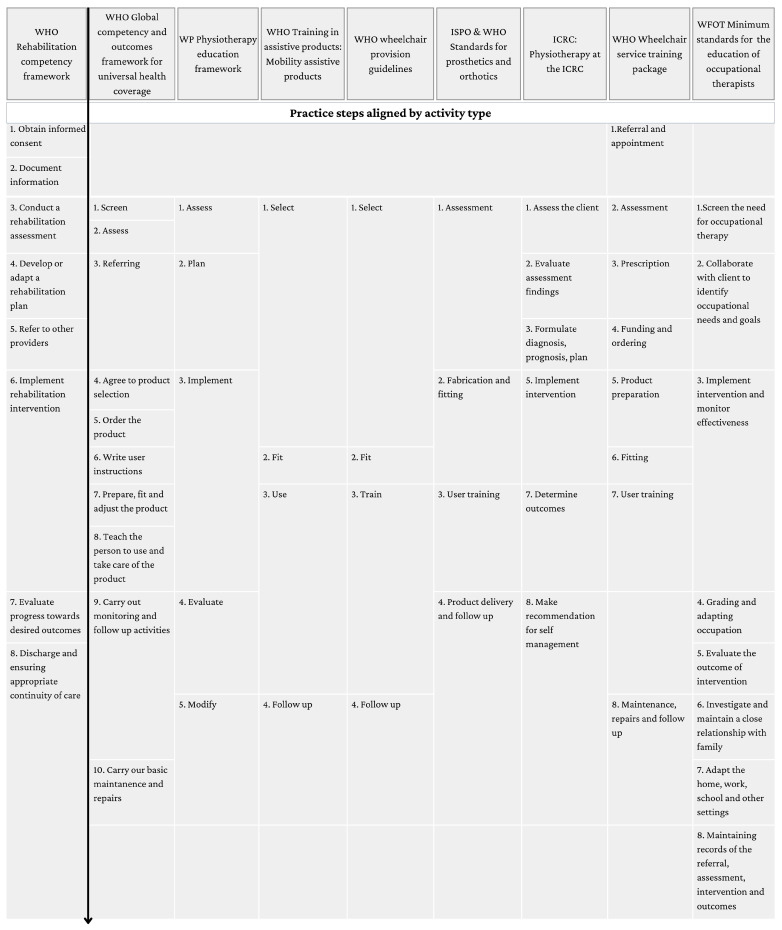
Practice steps contained within eight identified resources, in comparison to the WHO Rehabilitation Competency Framework. The arrow indicates the sequential nature of the practice steps.

Eight identified resources listed the practice activities/service steps involved in the provision of services including mobility assistive products (Figure 2). Across these resources, several common elements emerged. All featured early initial assessment phases, such as “Screen” (Global Competency Framework), “Assess the Client” (ICRC), Assess (WP), and “Referral and appointment” (WHO-Wheelchair service training package). In the subsequent steps, planning and implementation were consistently highlighted. The WHO RCF included “Develop or Adapt a Rehabilitation Plan” and “Implement Rehabilitation Intervention”. Similarly, the WP Physiotherapy Education Framework detailed “Plan” and “Implement”, while the ICRC framework included “Formulate Diagnosis, Prognosis, Plan” and “Implement Intervention”. The Wheelchair Service Training Package also outlined detailed steps, including “Prescription”, “Funding and Ordering”, and “Product Preparation”.

Follow-up and continuity of care were also recurring themes. The WHO RCF’s “Discharge and Ensuring Appropriate Continuity of Care” paralleled the WHO Wheelchair Provision Guidelines’ and WHO Training in Assistive Products’ emphasis on “Follow Up”. Technical and practical aspects were more pronounced in certain frameworks. The WHO Training in Assistive Products and WHO Wheelchair Provision Guidelines included “Fit”, “Use”, and “Train”, focusing on the hands-on provision and training aspects. The ISPO and WHO Standards for Prosthetics and Orthotics emphasize “Assessment”, “Fabrication and Fitting”, and “User Training”. 

## 4. Discussion

This environmental scan identified several global resources relating to competencies for mobility assistive products. Resources were both profession-specific and multidisciplinary and included education standards, training resources, service guides, and frameworks. The resources contained information about the competencies required to provide mobility assistive products and other details like the service steps and minimum education level. These findings suggest that organizations at different levels and in different sectors are working to improve assistive technology services which is positive; however, there has been a lack of linkage between initiatives thus far. 

Compounding the lack of linkage between existing resources is the absence of resources for several key professions identified as mobility assistive product providers in various global documents [1,10,24]. Community health workers, nurses, and medical professionals are identified as providers of mobility assistive products [1]. However, there are no global resources for these professions that outline relevant competencies specific to mobility assistive products. This raises the question of whether these professions consider assistive product provision within their scope of practice, an extended scope activity, or alternatively, as skills learned on the job. Engaging with these professions is essential to develop a comprehensive core set of competencies, ensuring all providers have the necessary skills and knowledge to deliver effective assistive technology services and provide mobility assistive products [1,14,17].

Similarities in service steps for mobility assistive product provision were identified in this research, a finding that is echoed in the recent scoping review by Layton et.al, 2024. Similar to our findings, this scoping review also identified congruency in the types and sequence of service steps [17]. Whilst the language used to describe each service step differed between resources, i.e., ‘follow up’ versus ‘ongoing monitoring’, they were largely referring to the same activities. Some resources contained more granular service steps than others, breaking down larger activities into smaller steps; however, they painted an overall similar picture of assistive technology services [17]. The lack of consistent language is also cited by Mills et al., in a scoping review of competency framework terminology [32]. 

There is a lack of global consensus on the specific competencies required for the provision of mobility assistive products, the professional cadres able to advise on or provide them, and the professional qualifications necessary to deliver them. The identified resources, while often comprehensive in their own right, varied significantly in their standards and requirements. Significant differences between competency domains were noted, for example. Furthermore, the profession-specific standards set minimum education requirements, whereas other resources referred to the entire ‘health workforce’ as potential mobility assistive product providers, regardless of their training or education level [23,31]. These discrepancies may lead to inconsistencies in practice, ultimately hindering access to high-quality mobility assistive products globally.

While some mobility assistive products may appear simple, such as walking sticks or manual wheelchairs, the complexity of their provision grows when considering the people who use them, in combination with the activities and the environments in which they are used [14,33]. Each individual mobility assistive product user presents a unique set of mobility goals, needs, circumstances, and potential complications, and the current research has further highlighted the need for a person-centered team approach [14]. Moreover, competencies for the provision of mobility assistive products must not only consider the technical features of the assistive product but also the advanced reasoning skills required to assess and address the nuances of each user. Federici et al. support this contention, emphasizing that it is often a series of well-reasoned assessments that is crucial for the mobility assistive product to achieve its purpose of reducing the mismatch between the person’s need and their environment and promoting well-being [34]. This level of complexity appears to be best captured by the standards and framework resources identified, but less so by the training resources. 

A further complication arises from the nature of the identified training resources, which tended to list specific activities required for assistive product provision, such as selecting and adapting, fitting, teaching the person to use the product, and following up [17,24]. Unfortunately, they often listed such activities in the absence of reference to a broader competency framework that integrates these activities into an underlying comprehensive skill set necessary for effective practice. This lack of a holistic approach may result in fragmented training that does not fully prepare professionals for the complexities of real-world service provision. For example, a basic walking stick may require adjustments based on the person’s height, weight, and gait pattern, as well as considerations of their living environment and daily activities. Failing to capture this complexity can lead to mismatched products, reduced effectiveness, product abandonment, and even harm to the person using the assistive product [35]. 

To promote safe and effective mobility assistive product provision and to allow for more synchronized efforts to grow the global assistive technology workforce, the WHO RCF could be adapted for the assistive technology sector as a fit-for-purpose competency framework; alternatively, an iterative Delphi process could be initiated with assistive technology stakeholders, as undertaken in several other professions including nursing and prosthetics and orthotics [12,36,37,38,39]. This development of a bespoke mobility assistive technology framework would serve as a critical resource for the wide range of professions that provide mobility assistive products, promote a globally uniform standard of service, and improve the overall quality of mobility assistive product provision worldwide [40].

Considering mobility assistive products are developing rapidly, the initiation of a mobility assistive product competency framework is particularly important and would promote that training resources go beyond listing isolated activities to include transferable skills. Transferable skills, like client assessment or collaborative goal settings, would apply not only to mobility assistive products available today, but also to any new product that comes to market. A competency framework could provide a comprehensive view that connects service activities with the underlying competencies required for holistic and effective practice, regardless of the product [10]. For example, while training might teach how to fit a mobility assistive product, a competency framework would ensure that the provider also has the communication skills needed to instruct the user on proper use and maintenance, cultural sensitivity to address diverse needs, and ethical considerations for equitable service provision. These competencies apply across mobility assistive product types [10].

### Limitations

While this research benefits from a comprehensive global search and direct contact with standards-setting professional organizations, the search was limited to documents available in the English language. This may have excluded relevant resources published in other languages, potentially missing valuable insights and competencies from non-English speaking regions [12]. 

## 5. Conclusions

People who use mobility assistive products need consistent high-quality services from their provider, regardless of profession. This environmental scan highlights a critical opportunity for professionals to collaborate to establish consensus on the competencies required for mobility assistive product provision. By defining these competencies, training, regulation, and service standards can be harmonized, enhancing coordination and service quality. This unified approach could also serve as a model for standardizing competencies for other assistive products, such as hearing and vision aids, improving outcomes across various assistive technologies. Establishing a core set of competencies by adapting the WHO RCF will ensure increased consistency in training and practice [10]. This will enhance access to safe and effective mobility assistive products worldwide.

## Figures and Tables

**Table 1:** Table of known global professional bodies that set standards for assistive technology professions.

Professional Groups	Global Representative Body
Assistive technology professionals	Global Alliance of Assistive Technology Organizations (GAATO)
Physiotherapy	World Physiotherapy (WP)
Prosthetics/orthotist	International Society of Prosthetics and Orthotics (ISPO)
Occupational Therapy	World Federation of Occupational Therapy (WFOT)
Medicine	World Medical Association (WMA)
Rehabilitation medicine	International Society for Physical Rehabilitation Medicine (ISPRM)
Nursing	International Council of Nurses (ICN)
Community health workers	1. Community Health Worker Central (CHW) 2. Community Health Impact Coalition (CHIC)
Wheelchair professionals	International Society of Wheelchair Professionals (ISWP)

**Table 2:** Included resources containing mobility assistive product competency information, organized by year of publication and resource type.

Organization(s)	Year	Resource Name	Profession(s) Described
**Service standard/guide**			
WHO	2023	Package of interventions for Rehabilitation Module 2: Musculoskeletal conditions Module 3: Neurological conditions	Rehabilitation professionals
WHO, ISWP, ISPO	2023	Wheelchair provision guidelines	Wheelchair professionals
WP	2011	Standards of physical therapy practice	Physiotherapy
WHO and ISPO	2017	WHO standards for prosthetics and orthotics	Prosthetic orthotic occupations
**Training resources**			
WHO	2022	Mobility assistive products—training in assistive products and subsequent modules: walking aids	Assistive technology workforce
Physiopedia	2022	Assistive technology: Mobility Products and steps to assistive technology provision	Health personnel
WHO and USAID	2012	Wheelchair Service Training Package- Basic and Intermediate	Wheelchair personnel
**Education standards**			
WP	2021	Physiotherapist education framework	Physiotherapy
ISPO	2017	Education Standards for Prosthetic and Orthotic Occupations	Prosthetic orthotic occupations
WFOT	2016	Minimum Standards for the education of occupational therapists	Occupational therapy
**Workforce framework**			
WHO	2023	Global competency and outcomes framework for universal health coverage	Health workforce
UNICEF	2022	Framework for building capacity for assistive technology and alternative augmentative communication for children	AT professionals, Professionals in Education, Health and Social Care
**Organizational manual**			
ICRC	2022	Physiotherapy within ICRC	Physiotherapy
**Profession profile/description and scope of practice document**			
WHO	2017	Human resources for medical devices, the role of biomedical engineers	Biomedical engineers

Table acronyms: WHO—World Health Organization, ISWP—International Society of Wheelchair Professionals, ISPO—International Society of Prosthetics and Orthotics, WFOT—World Federation of Occupational Therapists, ICRC—International Committee of the Red Cross, WP—World Physiotherapy, USAID—United States Agency for International Development, UNICEF—United Nations International Children’s Emergency Fund.

**Table 3:** Identified resources that contain competency domains.

WHO	WP	WP	WFOT	ISPO
Global Competency and Outcomes Framework for Universal Health Coverage	Standards of Physical Therapy Practice	Physiotherapist Education Framework	Minimum Standards for the Education of Occupational Therapists	Education Standards for Prosthetic and Orthotic Occupations
6 Domains	10 Domains	8 Domains	14 Domains	10 Domains
1. People-centredness, 2. Decision-making, 3. Communication, 4. Collaboration, 5. Evidence-informed practice and 6. Personal conduct	1. Administration and practice 2. Communication, 3. Community Responsibility 4. Cultural competence 5. Documentation 6. Education 7. Ethical behavior 8. Informed consent 9. Legal 10. Patient/client management	1. Physiotherapy assessment and intervention 2. Ethical and professional practice 3. Communication 4. Evidence-based practice 5. Interprofessional teamwork 6. Reflective practice and lifelong learning 7. Quality improvement 8. Leadership and Management	1.The person-environment-occupation relationship and its relationship to health, well-being and human rights 2. Environment 3. Relationship between occupation and health, well-being and human rights 4. Therapeutic and professional relationships 5. Relationships with recipients of occupational therapy 6. Relationships with team and organizational members 7. OT Process 8. Health and social systems and service delivery models 9. Research/information search process 10. Ethical practice 11. Professional competence 12. Reflective practice 13. Managing self and others 14. Contexts of professional practice	1. Apply knowledge of physical sciences, social sciences, health sciences, culture, and natural sciences to professional practice 2. Demonstrate proficiency in communication skills 3. Participate in the development of practice management skills in various settings 4. Work effectively in an inter/intra-professional collaborative setting 5. Demonstrate social and professional responsibility and ethical behaviors in multi-cultural settings and scenarios 6. Demonstrate competence in conducting examination, evaluation, and assessment of users across the individual’s lifespan and within a broad continuum of care 7. Optimize the use of equipment, materials, components and techniques in prosthetic/ orthotic services. 8. Demonstrate competence in developing and implementing prosthetic/orthotic service plans for users across the individual’s lifespan within a broad continuum of care 9. Demonstrate, in a systematic and effective manner, the ability to impart knowledge when providing education for users, their caregivers, other health professionals, and the public at large 10. Demonstrate competencies in research and evidence-based practice

## Data Availability

All relevant data have been supplied in the article.

## Appendix A

**Table A1:** Classes and subclasses of assistive products related to mobility assistive products from ISO 9999 Assistive products—classification and terminology (2022) [11].

Class	Subclass
Class 6: Orthoses and Prostheses	06 12: Lower limb orthoses 06 24: Lower limb prostheses
Class 12: Assistive Products for Activities and Participation Relating to Personal Mobility and Transportation	12 03: Assistive products for walking, manipulated by one arm 12 06: Assistive products for walking, manipulated by both arms 12 07: Accessories for assistive products for walking 12 08: Guide canes and symbol canes for orientation 12 10: Cars, vans, and pick-up trucks 12 11: Mass transit vehicles 12 12: Vehicle accessories and vehicle adaptations 12 16: Mopeds and motorcycles 12 17: Diverse motorized vehicles 12 20: Cycles 12 22: Manual wheelchairs 12 23: Powered wheelchairs 12 24: Wheelchair accessories 12 25: Accessories for wheelchair seating 12 27: Diverse human-powered vehicles 12 31: Assistive products for changing body position 12 36: Assistive products for lifting persons

## Appendix B

**Table A2:** Search strategy.

Search Number	Search Field	Search Terms
1	Kw ^1^, aip ^2^	Global or international
2	Kw, aip	alliance OR association OR union OR council OR federation OR group OR organization
3	Kw, aip	standard OR framework OR competenc*
4	Kw, aip	assistive technolog* OR assistive product*
5	Kw, aip	Physio* OR physical therap* OR prosthe* OR orthot* OR occupational therap* OR nurs* OR doctor OR physician OR medica* OR community health OR assistant OR technician OR orthopaedic tech*
6		1 AND 2 AND 3 AND 4 AND 5
**Other search parameters**	Language	English
	Region	Any region
	File type	All formats (.doc, .docx, .txt, .xls, .xlsx, .ppt, .pptx, .rtf, .ps)
	Last usage	Anytime
	Usage rights	Publicly available, open access
	Results per page	Set to 100
	Publication year	Unrestricted
	Version	Where multiple versions or iterations are found, the most recent version will be included only

^1^ Keyword. ^2^ anywhere in page, including title, text, links, and URL. * indicates a wildcard, allowing for multiple variations of a word ending to be identified in the search.
